# Chromonomer: A Tool Set for Repairing and Enhancing Assembled Genomes Through Integration of Genetic Maps and Conserved Synteny

**DOI:** 10.1534/g3.120.401485

**Published:** 2020-09-10

**Authors:** Julian Catchen, Angel Amores, Susan Bassham

**Affiliations:** * Department of Evolution, Ecology, and Behavior, University of Illinois at Urbana-Champaign, IL; † Institute of Neuroscience, University of Oregon, Eugene, OR; ‡ Institute of Ecology and Evolution, University of Oregon, Eugene, OR

**Keywords:** Genome assembly, genetic map, RADseq, conserved synteny

## Abstract

The pace of the sequencing and computational assembly of novel reference genomes is accelerating. Though DNA sequencing technologies and assembly software tools continue to improve, biological features of genomes such as repetitive sequence as well as molecular artifacts that often accompany sequencing library preparation can lead to fragmented or chimeric assemblies. If left uncorrected, defects like these trammel progress on understanding genome structure and function, or worse, positively mislead this research. Fortunately, integration of additional, independent streams of information, such as a marker-dense genetic map and conserved orthologous gene order from related taxa, can be used to scaffold together unlinked, disordered fragments and to restructure a reference genome where it is incorrectly joined. We present a tool set for automating these processes, one that additionally tracks any changes to the assembly and to the genetic map, and which allows the user to scrutinize these changes with the help of web-based, graphical visualizations. Chromonomer takes a user-defined reference genome, a map of genetic markers, and, optionally, conserved synteny information to construct an improved reference genome of chromosome models: a “chromonome”. We demonstrate Chromonomer’s performance on genome assemblies and genetic maps that have disparate characteristics and levels of quality.

Researchers are generating new reference genomes at an accelerating pace. While it is now straightforward to produce enough sequence information to cover even large genomes many times over, the assembly of a realistic reference genome can still be challenging for both bioinformatic and biological reasons (Church *et al.* 2011; De La Torre *et al.* 2014; Nowoshilow *et al.* 2018; Ghurye and Pop 2019). A high-quality reference genome with minimized gaps and misassemblies, particularly one organized into chromosomes – known as a *chromonome* (Braasch *et al.* 2015) – is a valuable research tool. Comparative genomics studies that have employed, for example, the analysis of conserved synteny of genes among distantly-related taxonomic groups have led to better understanding of how genes and genomes evolve and function (Naruse 2004; Jaillon *et al.* 2004; Shah *et al.* 2012; Lovell *et al.* 2014; Zhao and Schranz 2019). Likewise, understanding the population dynamics of selection and drift, as described by measures of mutation and linkage, requires chromosome-level stretches of sequence (Hohenlohe *et al.* 2010; Luikart *et al.* 2018). Reliably assembled reference genomes have aided exploration of chromosome structural conservation or rearrangement through evolutionary time (Wang *et al.* 2013; Jay *et al.* 2018), the effects of transposable element perturbation (Woronik *et al.* 2019), the fate of duplicated genes following divergence of organismal lineages (Brunet *et al.* 2006; Kassahn *et al.* 2009), the mechanisms of long distance regulation of genes (Kleinjan and van Heyningen 2005), and the progression of disease-resistant alleles in populations (Epstein *et al.* 2016). New reference genomes that are misassembled, or that remain broken in scaffolds, or whose scaffold order relies only on the reference genome of a different taxon, can stall or mislead inferences about critical biological processes.

Sequencing technologies and genome assembly strategies continue to evolve, but it is still not trivial to assemble chromosome level references with highest confidence in biological accuracy for organisms with complex genomes. Since the inception of high-throughput sequencing three major assembly strategies have been employed: short-read-only assemblies, hybrid assemblies that incorporated long reads to join and gap-fill short-read assemblies, and long-read-only assemblies. While contig generation has become very robust, whether it is via the use of a *de Bruijn* graph in short-read and hybrid assemblies (Compeau *et al.* 2011), or through the use of polishing algorithms in long-read assemblies (Fu *et al.* 2019), most obstacles to the generation of a chromonome come from the error models of different scaffolding approaches.

Short-read assemblies rely on incorporation of “mate-pair” sequences to order and orient contigs into scaffolds (Gnerre *et al.* 2011; Chapman *et al.* 2011; Luo *et al.* 2012), but the approach can produce molecular chimeras during library construction or assembly chimeras during scaffolding when the short reads land in repeats. Optical maps (Pendleton *et al.* 2015; Howe and Wood 2015) and chromosomal conformation capture methods such as Hi-C (Lieberman-Aiden *et al.* 2009) have been used very effectively for scaffolding and have improved assembly quality metrics like N50 and L50. These long molecular methods are not immune from errors, however, which manifest as indels and fragment length estimation mistakes (Mukherjee *et al.* 2018), artifactual inversions, and occasional long-range chimeras during integration into an assembly (Ghurye *et al.* 2019). While all scaffolding methods remain imperfect, independent methods to explore and verify genome organization remain valuable. A genetic map is a multifunctional tool that can also serve this purpose.

Genetic map construction remains relevant for a variety of research goals; for example, comparing a genetic map with a physical genome sequence helps identify gene candidates causal for variant or mutant phenotypes (Meinke *et al.* 2003; Peichel and Marques 2017), and reveals variation in recombination rate across the genome (Roesti *et al.* 2013; Dukić *et al.* 2016). A map can also benefit the assembly of a reference genome by revealing points of erroneous contiguity in an assembly, by binding scaffolds into “linkage groups” that are chromosome models, and by ordering and orienting the scaffolds relative to one another. It is now relatively straightforward and rapid to genotype individuals at thousands of loci by using one of many massively parallel sequencing methods such as Restriction site-associated DNA sequencing (RADseq; (Baird *et al.* 2008; Davey *et al.* 2011; Andrews *et al.* 2016)). Marker-dense maps have the potential to capture a majority of the assembled genome length into linkage groups. More importantly, potentially chimeric scaffolds can be detected where the physical and genetic map relationships of markers on scaffolds conflict, such as in cases where a single scaffold’s markers map to more than one linkage group. The efficacy of a genetic map for consolidating an assembly and validating its quality depends on a number of important factors, including the density and distribution of markers, the number of meiotic crossovers represented in the mapping cross progeny, the size distribution of the scaffolds, the granularity of misassembly with respect to the distance between markers, and the genotyping accuracy.

In the end, a genome assembly is a hypothesis that proposes a sequence order while the true order will always remain unknown. A useful tool should be able to automate the flagging of problematic scaffolds, resolve conflicts between the assembly and the genetic map in a rational and efficient way, and integrate additional lines of evidence that support a hypothesis of genomic structure. We present here software we call Chromonomer that corrects, orders, and orients scaffolds by integrating genetic maps and genome assemblies. Chromonomer can create chromosome-level assemblies while providing extensive documentation of how the elements of evidence fit together. To further improve assemblies, the software can integrate conserved gene synteny and raw read depth of coverage, and it provides tools to extract gene annotations from a scaffold-level assembly and translate their locations to a chromosome-level assembly (and vice versa). Earlier, prototype versions of Chromonomer have been used in a number of published genome assembly integrations (*e.g.*, Amores *et al.* 2014; Fountain *et al.* 2016; Small *et al.* 2016; Kim *et al.* 2019; Moran *et al.* 2019). Here we illustrate the performance of Chromonomer with three qualitatively different teleost genome test cases representing the three major assembly strategies: 1) a high-quality, short-read-based assembly with a map made from a modestly sized genetic cross (Gulf pipefish), 2) a high-quality, long-read, optical map-scaffolded assembly with a large genetic cross (platyfish), and 3) a highly scrambled, hybrid assembly with a large genetic cross (Antarctic black rockcod).

## Materials and Methods

The primary design goal of Chromonomer is to integrate disparate information (contigs, scaffolds, and genetic maps) in a hierarchy of reliability. In cases where the first source of information is ambiguous, the software can apply additional sources. Chromonomer is designed first to trust contiguous genome assembly, in other words, the *contigs*, where scaffolding has not yet been inferred from other molecular information. Next, Chromonomer trusts the overall linkage map ordering, followed by the scaffolding, raw read depth of coverage, and finally, conserved gene synteny, depending on what information is available and on the user’s dictate. Given this hierarchy of information, the Chromonomer algorithm 1) inserts virtual gaps into scaffolds, if depth of coverage data are supplied, 2) breaks inter-linkage group scaffolds using real or virtual gaps, 3) models each linkage group as a graph with scaffolds attached to graph nodes, 4) finds a consistently ordered set of markers, 5) breaks intra-linkage group scaffolds that span non-adjacent map nodes, and optionally, 6) orders and orients any unordered scaffolds using conserved gene synteny.

### The basal Chromonomer algorithm

Chromonomer requires a description of the genome assembly, which consists of an AGP (*A Golden Path*) file (NCBI 2019) describing the structure of the scaffolds (the set of ordered and oriented contigs and gaps), a tab-separated file describing the genetic linkage map, including the linkage group and centiMorgan (cM) position of each marker, and a SAM or BAM file (SAM/BAM Format Specification Working Group 2019) describing the alignment positions of the markers in the physical assembly. Optionally, a FASTA file containing the genome sequence can also be supplied (and with it, Chromonomer will provide a reordered FASTA file of physical sequence after the Chromonomer algorithm completes). The contig, scaffold, and marker IDs must match among the input files.

### Inter-linkage group conflicts

**Figure 1:**
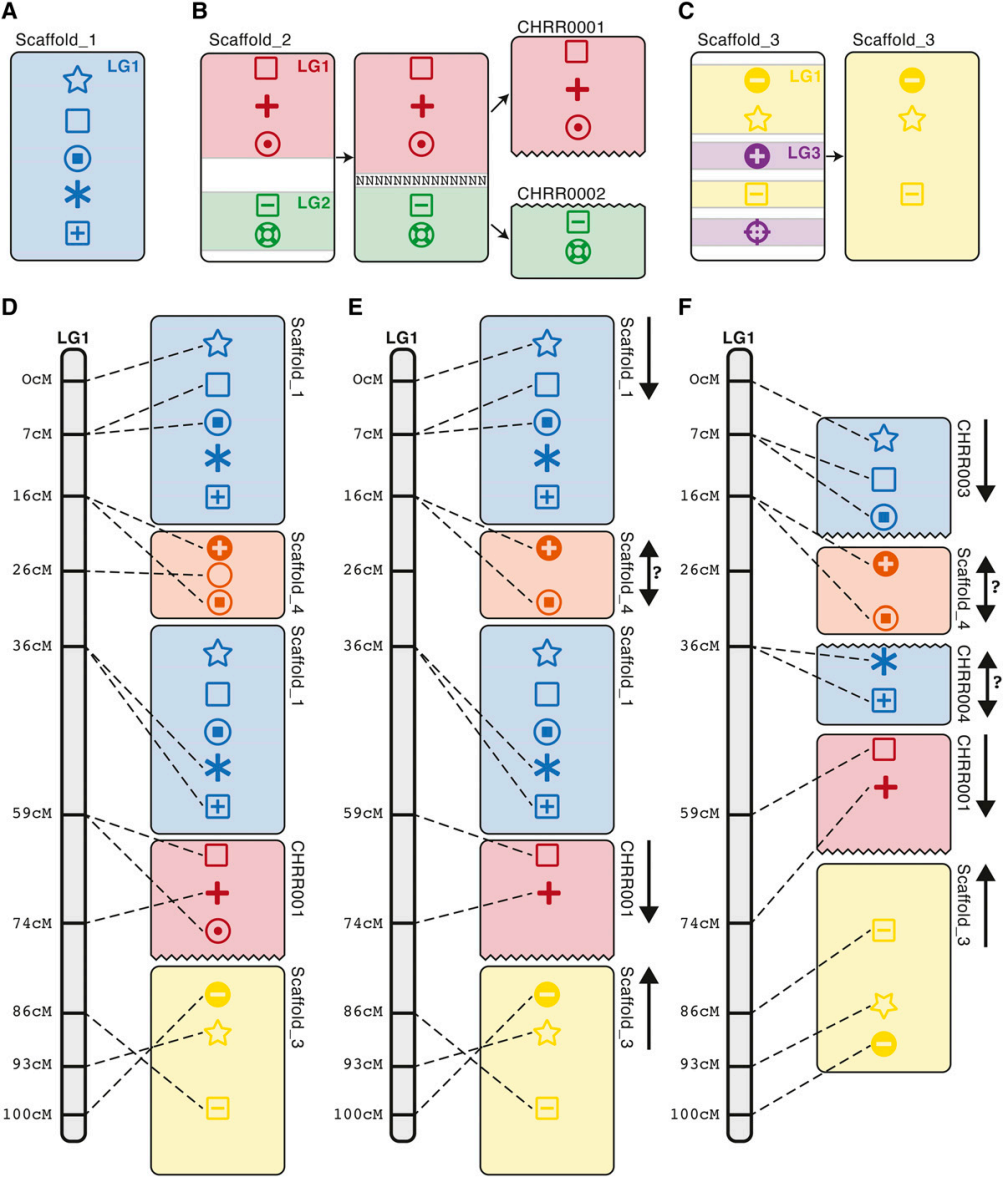
The primary Chromonomer algorithm. The algorithm takes a set of scaffolds (seen here as rectangles), a set of markers (typically DNA sequence, *i.e.*, RAD markers; represented here as shapes within the rectangles), an assembly file (AGP file), which describes how contigs and gaps are formed into scaffolds in the assembly, and a genetic map, which provides order to the markers. (A-C) Scaffolds are first evaluated to identify sets of markers mapped to different linkage groups. Those scaffolds will be split at the nearest gap (B) or pruned out (C) if a consistent set of markers cannot be found. (D) Scaffolds are anchored to their positions in the genetic map; if a scaffold appears in two locations in the genetic map, it is anchored twice. (E) A consistent ordering of markers is determined, with inconsistent markers discarded. (F) Scaffolds are oriented or split at the nearest gap, as dictated by the genetic map.

In the first stage of the algorithm, Chromonomer resolves *inter-linkage group conflicts*. For each scaffold, Chromonomer collects the markers aligned to it and sorts the markers by linkage group (Figure 1A-C). Since linkage group assignment is statistically very robust, the linkage map is trusted over the physical scaffolding. So, if markers on a single scaffold belong to more than one linkage group, Chromonomer will attempt to split the scaffold (Figure 1B). To do so, the markers must be in two or more consistently ordered sets, with an available scaffold gap (sequence of ‘N’ characters) between them; if multiple gaps exist, the largest gap is chosen. If such a configuration is not available, Chromonomer will discard sets of neighboring markers, starting with the smallest set, (Figure 1C) until the scaffold can be split, or until a single, consistent set of markers remain. Split scaffolds are renamed in a user-definable way, and the details of the process are logged.

Chromonomer next determines a provisional orientation for each scaffold by calculating a linear regression between the linkage map cM positions, and scaffold-aligned basepair positions of the inclusive markers. Although not all markers are consistently ordered yet, Chromonomer will orient the scaffold in the forward direction if a positive regression results, or in the reverse direction in the case of a negative regression. This requires markers to link a scaffold to at least two cM positions in the map.

### Modeling linkage groups as graphs

The Chromonomer basal algorithm represents each cM position in the map as a node in a graph. Markers are used to anchor scaffolds to their respective nodes in the graph; if a scaffold spans consecutive nodes, the nodes are collapsed together providing a definitive orientation for the scaffold. If a scaffold is anchored to multiple, non-neighboring nodes, it is placed into the graph in every position where at least one of its aligned markers occurs (Figure 1D). If multiple scaffolds collapse into the same, single graph node, their order (linear series) within the node cannot be determined from the map alone, though this cluster of scaffolds can still be ordered relative to scaffolds anchored to other nodes.

### Finding a consistent set of markers

Unlike how Chromonomer prioritizes map structure over scaffolding, the algorithm trusts the contiguous physical assembly over individual markers that are not corroborated by other, nearby markers – since genotyping errors can slightly change the position of a particular marker in the map. For each occurrence of a scaffold within a linkage group graph, Chromonomer will identify a maximal set of markers for the associated node that have a consistent order with respect to each other (marker base pair positions increase with map cM position, or the orders are inverted if in reverse orientation) (Figure 1E). The markers whose order conflicts with respect to each node are logged and discarded.

### Resolving intra-linkage group conflicts

Chromonomer next looks at each scaffold individually within the linkage group. Scaffolds that remain in multiple nodes of the graph indicate assembly errors. Since the markers that remain are consistently ordered, Chromonomer can break scaffolds at the nearest gap between the two groups of markers from each subset of the scaffold anchored to different graph nodes (*e.g.*, Scaffold_1 in Figure 1E and 1F), and the details of each split are logged. If an appropriate gap cannot be found to split the scaffold, the smallest set of markers at a particular graph node are discarded until the scaffold can be split across nodes, or until there is only one set of consistent markers left in a single graph node, which places the unsplit scaffold in a single location.

Finally, Chromonomer recalculates the orientation of each scaffold, again using linear regression of marker positions, and summarizes the new, chromonome-level assembly, creating a new set of sequences reflecting any scaffold splits (output in a FASTA file) and an assembly description (output in a set of AGP files) to describe the changes. An external script, translate_gtf.py, is provided to lift over a set of gene annotations from a scaffold-based assembly to a chromonome, or vice versa.

### Depth of coverage and virtual gaps

**Figure 2:**
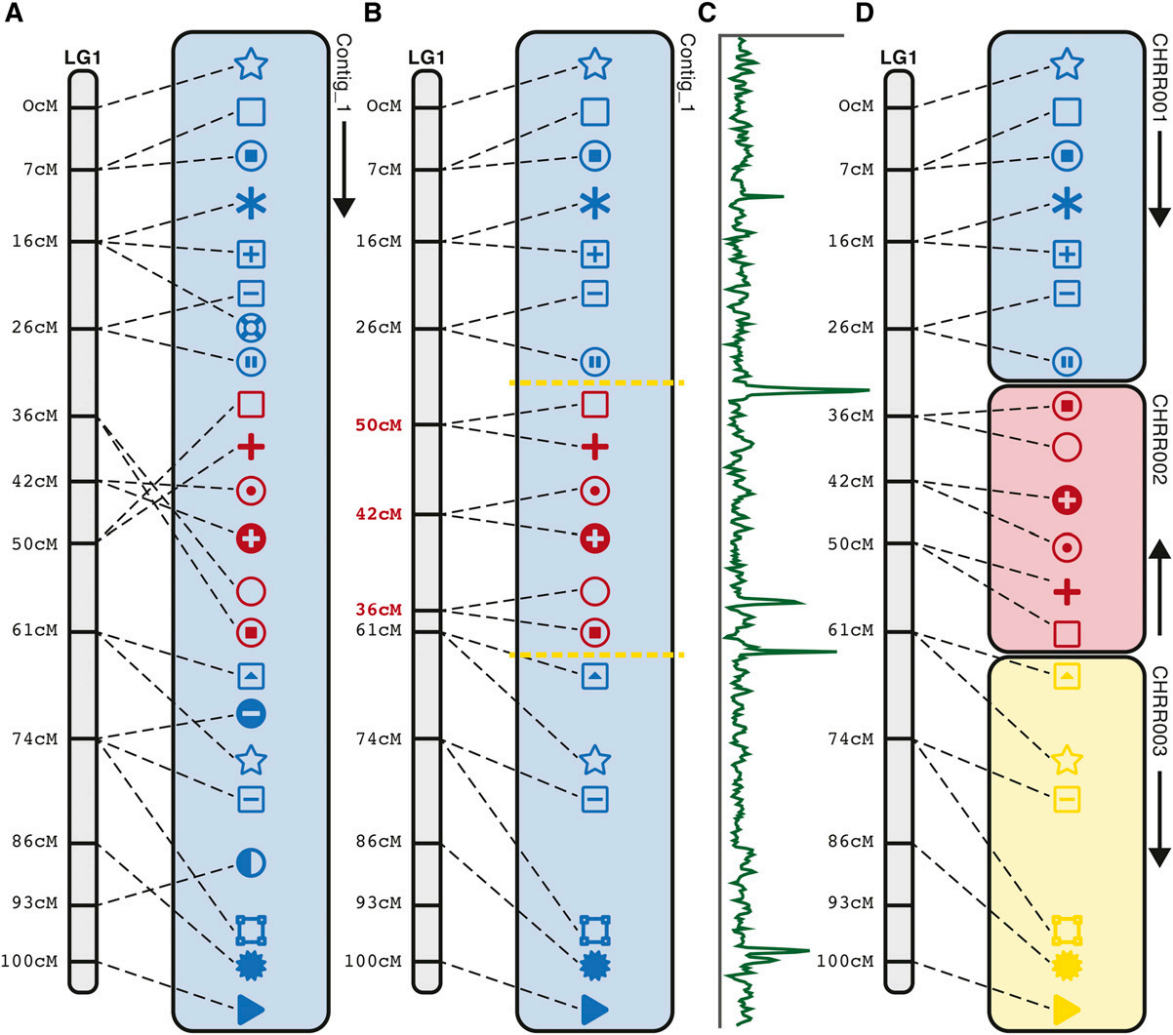
Using depth of coverage to create virtual gaps, and rescaffolding the assembly. Assemblies constructed using long reads often consist purely of contigs, with no gaps. In these cases, we can input into Chromonomer depth of coverage data, generated by aligning raw reads back to the assembly, and we can identify anomalous values in depth of coverage to direct where to create virtual breakpoints in the assembly. Here, in (A) the markers clearly show a misassembly in the center region of the contig (red markers). With no gaps, the normal algorithm to split the contig will fail. (B) The scaffolding algorithm instead assumes the genetic map is the correct source of information and identifies where the contig should be broken, according to a consistent set of reordered map markers. Depth of coverage information (C) is incorporated to identify logical break points and (D) the contig is split into the respective pieces.

Chromonomer relies on assembly gaps to break a scaffold when the genetic map indicates a misassembly. However, depending on the assembly process, a genome might be structured with very few, or without any, gaps. The alignment of raw reads back to the genome can reveal regions of anomalous depth, which are likely candidates for points of misassembly. Chromonomer can use per-base pair depth of coverage data, generated by samtools (Li *et al.* 2009), to identify these regions (Figure 2). Along each scaffold, Chromonomer slides a window (user definable, default 5Kbp) and calculates the mean depth within each window. It then determines how many standard deviations any window is from the scaffold depth mean. If a window is above or below the user-definable number of deviations (default is 3), a virtual gap of zero length is inserted at the 5′ end of the window into the internal AGP representation of the scaffold, which makes it available for Chromonomer’s standard splitting algorithm. Any virtual gaps not used during processing will be removed before outputting modified AGP or FASTA files.

### Ordering scaffolds with conserved gene synteny

If a scaffold does not span more than one cM node in the linkage group, it cannot be oriented by the map. Likewise, if two or more scaffolds are anchored to a single map node, they cannot be unarbitrarily ordered within that node. For these classes of scaffolds and only these, Chromonomer can be instructed to use the order of orthologous genes from a related genome to further specify order and orientation. In other words, conserved synteny data are subordinate to map location data. The user specifies the gene annotation of a related genome (in GFF or GTF format), the annotation of the focal genome at a scaffold level, and the orthology assignment of genes between the two annotation files (using a tab-separated file).

First, for each linkage group, the overall relative orientation of the “external” chromosome (*i.e.*, from the related genome) must be determined. To do this, Chromonomer calculates the regression of gene positions between definitively oriented scaffolds on the linkage group (that is, existing on two or more nodes in the graph) and the orthologous genes on the external chromosome. Based on this comparison, the orientation of the external chromosome is reversed if necessary.

Next, for each node in the linkage group that contains more than one scaffold (that is a node in the map that hosts unordered or unoriented scaffolds), Chromonomer tabulates the genes present on the collection of scaffolds. Any genes that fall on a non-orthologous external chromosome and any singleton, out-of-order genes are excluded, and the set of genes with a congruent order is retained. Similarly, orthologous genes that are too far away from the main set of orthologs, as determined using a trimmed mean algorithm (Bednar and Watt 1984), are discarded. The genes on the set of scaffolds are ordered according to the external chromosome position, which then allows ordering of the scaffolds in the node. Finally, the orientation of each scaffold is determined independently by calculating a regression based on the basepair positions of the genes on the scaffold and the external chromosome.

### Rescaffolding based on the genetic map

The *rescaffold* algorithm provides a way to rationally break gapless contigs that the basal algorithm alone cannot resolve, by reprioritizing the map marker order over the assembly contig boundaries (Figure 2). Each node in the linkage group graph will be considered the ‘owner’ of the sequence that ‘its’ markers span. Because genotyping errors can shift marker order in the map relative to the physical sequence, sets of markers from adjacent map nodes must be found that do not overlap in the physical sequence. The first step is to bucket sets of markers according to their graph node origin and to calculate a mean basepair position to represent the map node in the physical sequence. Next, the set of nodes (and their markers) are re-sorted according to these mean basepair positions. Then adjacent nodes are traversed, and the algorithm prunes any markers whose basepair positions overlap between the current and next nodes (Figure 2A). Markers are pruned in rounds, according to how far a marker is from the mean basepair position for the map node. This has the effect of removing markers that are the farthest away in the physical sequence from the mean map node position first. Pruning continues until no markers overlap between the nodes. Breakpoints are required for the algorithm to proceed further, and if the sequence in the integration has no gaps, Chromonomer can insert virtual gaps via raw read depth of coverage (Figure 2B, yellow lines). The contig is finally broken into pieces using the basal algorithms described above, and after splitting, the map nodes are re-sorted back to their cM positions (Figure 2D); this constitutes the reordering and reorienting of the new components of the broken contig.

### Chromonomer outputs

Chromonomer creates ‘before’ and ‘after’ log files for each linkage group; it creates a specific log for each modified scaffold. These logs detail marker positions relative to the genetic map and their genomic alignments. Markers dropped due to conflicts are shown and a reason for dropping them supplied. General statistics, such as integrated chromosome lengths, and the location and number of splits are logged. The software also computes a list of “promising scaffolds”: scaffolds that could be integrated into the linkage groups by improving the map. Chromonomer will also produce FASTA, AGP, and GFF files that describe the newly integrated assembly.

### Web-based visualization

**Figure 3:**
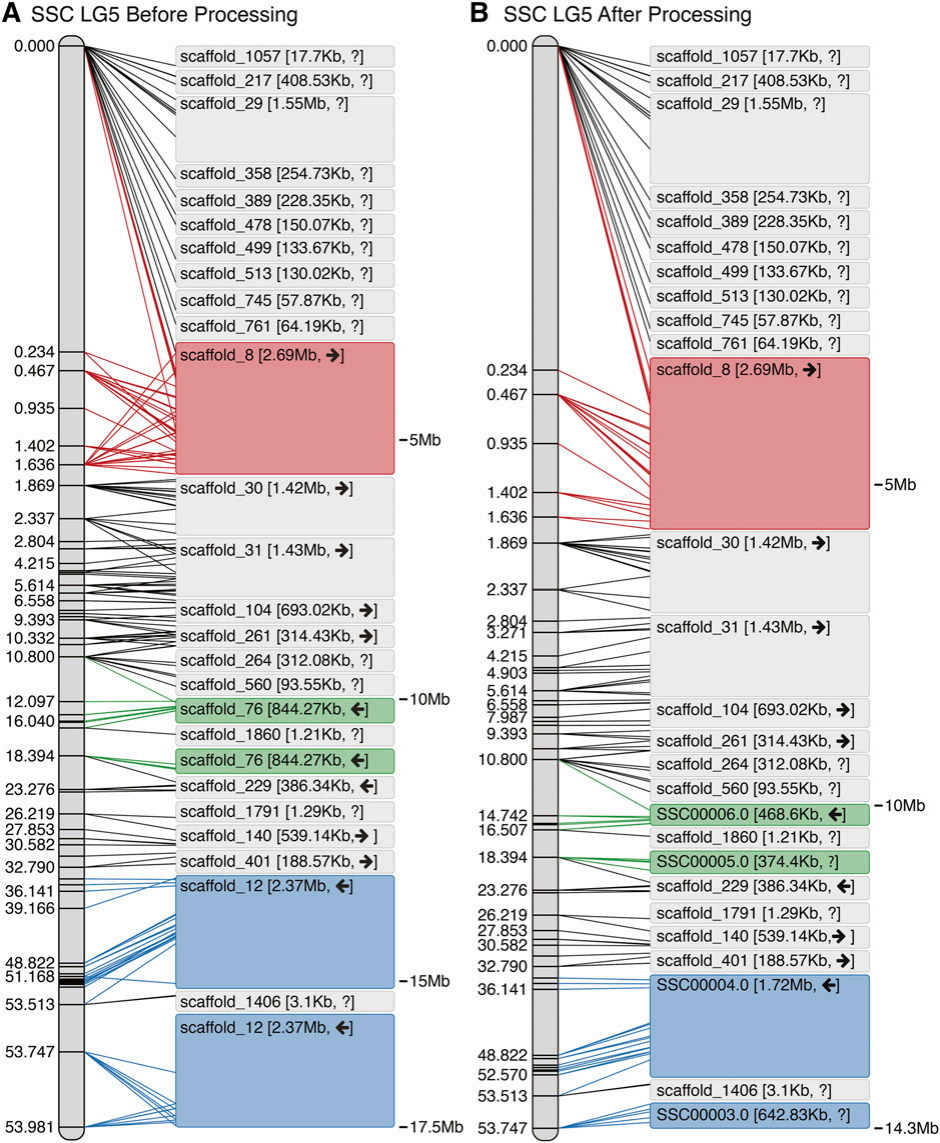
The Chromonomer algorithm as employed in the Gulf pipefish (*Sygnathus scovelli*) assembly. The figure shows all the numbered scaffolds belonging to LG5 before (A) and after (B) processing. In the diagrams, each marker in the linkage group (left) is connected by a line to its alignment position on each scaffold (right). In red in (A), scaffold_8 demonstrates markers with conflicting physical and map orders. In (B), the order of markers has been resolved and some conflicting markers discarded. Scaffold_76 (green) and scaffold_12 (blue), which are each anchored in two map positions, demonstrate examples of scaffolds that need to be split so a third scaffold can be inserted into the rift.

**Figure 4:**
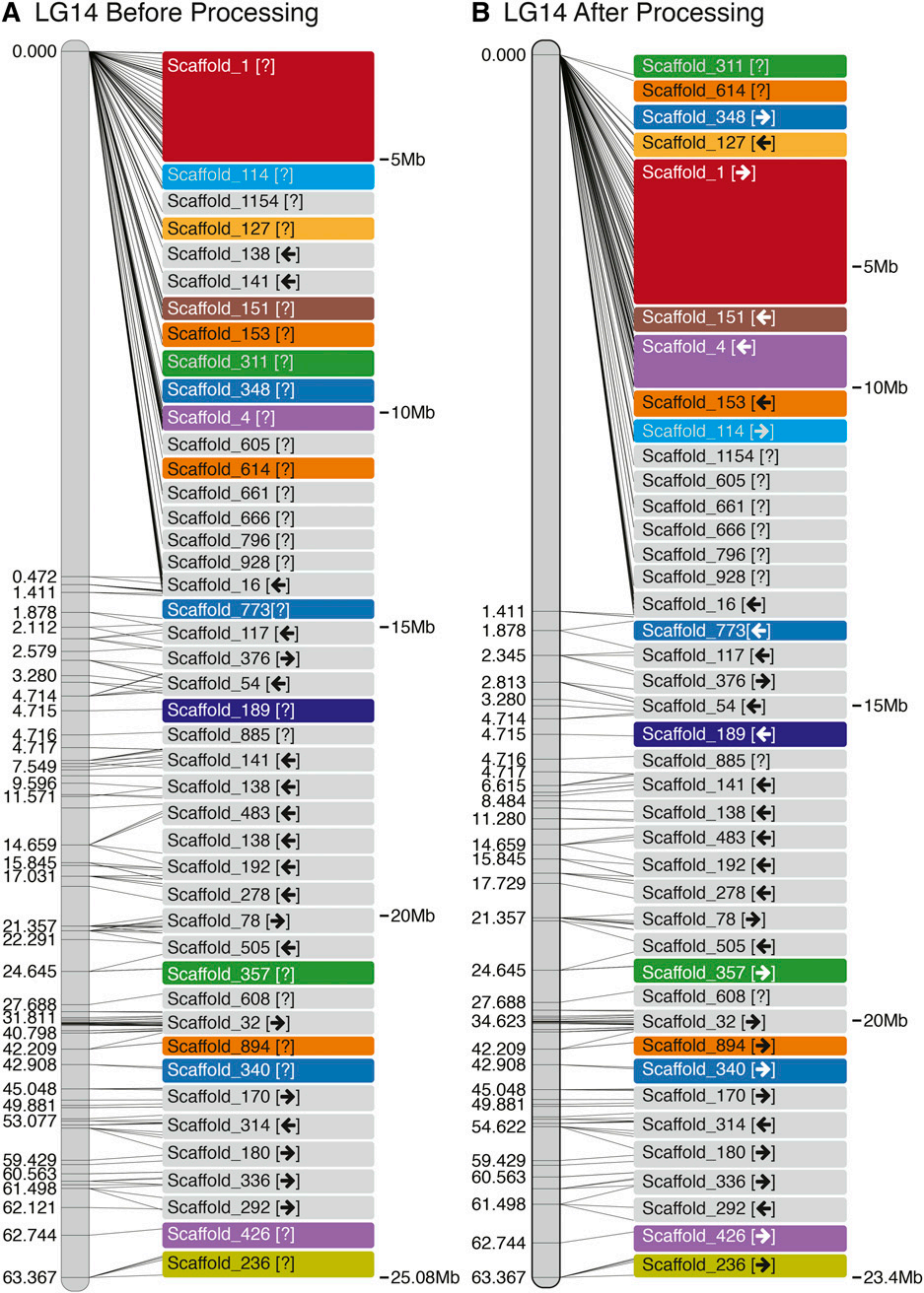
Including conserved gene synteny into the Chromonomer algorithm, as employed in the Gulf pipefish (*Sygnathus scovelli*) assembly. The figure shows LG14 before (A) and after (B) processing. In this example we have incorporated conserved gene synteny from the close relative *Sygnathus acus* to order and orient scaffolds whose position and orientation are left ambiguous by the genetic map. Colored scaffolds indicate where synteny was employed.

For each run of Chromonomer, the output directory of data can be made visible to a web server (*e.g.*, Apache, not supplied) and then served via HTTP. Chromonomer pre-computes a ‘before’ and ‘after’ JSON file (JavaScript Object Notation; https://tools.ietf.org/html/rfc8259) for each scaffold. These JSON files are used to create graphical visualizations of the ‘before’ and ‘after’ states of the linkage groups that can be viewed in a web browser. Figures 3, 4, and 6 are based on these visualizations. Chromonomer includes JavaScript code that will also be served by the web server to render these visualizations in the web browser using the D3 library (https://d3js.org/). If a web server is not available, textual versions of these visualizations are also supplied.

### Genome integrations

The description of Chromonomer genomic analyses, including commands executed for Gulf pipefish, platyfish, and rockcod genomic integrations can be found in the Supplementary Methods.

### Data availability

All input data were previously available in online repositories and the appropriate accession numbers are listed inline in the Supplementary Methods section. The three integrated genomes have been deposited in the Dryad Data Repository at https://doi.org/10.5061/dryad.gtht76hjm. Chromonomer is released as open source software, under the GPL v3 license. Documentation can be found, and the source code may be downloaded from http://catchenlab.life.illinois.edu/chromonomer. Chromonomer can be built on UNIX-like systems (*e.g.*, Linux and MacOS) and has no mandatory dependencies on other software. Chromonomer can be executed on any modestly capable computer (laptop or server). Supplemental material available at figshare: https://doi.org/10.25387/g3.12837545.

## Results

### The basal Chromonomer algorithm: the Gulf pipefish genome

Chromonomer was used to integrate the physical assembly of the Gulf pipefish (*Sygnathus scovelli*) with a genetic map derived from an F1 cross of 108 progeny (Small *et al.* 2016). This reference genome is an Illumina-based assembly following the ALLPATHS-LG assembly strategy (see Table 1 for details). The map consisted of 6593 markers on 22 linkage groups while the physical assembly was 307Mbp in total length, contained in 2104 scaffolds, and had a scaffold N50 of 640Kb and an L50 of 115.

**Table 1:** Characteristics of assembled genomes integrated with Chromonomer

Organism	Assembly Strategy	Scaffold Count	Scaffold N50	Assembly Size	Scaffolds Integrated	Length Integrated
**Gulf pipefish (*Sygnathus scovelli*)**	-2x180bp Illumina reads	2,104	0.64Mbp	307Mbp	550 [555 after splitting]	87%
	-Illumina mate-pair reads					
	-ALLPATHS-LG assembler					
**Southern platyfish (*Xiphophorus maculatus*)**	-PacBio Sequel I	101 [24 chrs.]	31.5Mbp	704Mbp	31 [117 after splitting]	99.5%
	-Bionano Optical Map					
	-HGap assembler					
**Antarctic bullhead notothen (*Notothenia coriiceps)***	-2x150,300,350,500,600bp Illumina reads	38,656	0.22Mbp	636Mbp	2,803 [3,847 after splitting]	63%
	-454 GS-FLX mate-pair reads					
	-PacBio RS II reads					
	-Canu assembler, Gapfiller, PBJelly scaffolders					

Figure 3 shows how the Chromonomer algorithm handled one linkage group. It is clear that prior to processing, marker order is inconsistent with alignment order (*e.g.*, see Figure 3A, in which red lines are crisscrossed), but after processing their order has been corrected by discarding incongruous markers (the red lines are resolved in Figure 3B). Scaffold 76 (Figure 3A, green) appears in the map twice, as does scaffold 12 (Figure 3A, blue). After processing (Figure 3B), both scaffolds have been split, and in each case an additional scaffold has filled a gap (scaffolds 1860 and 1406, respectively). After Chromonomer integrated the genetic map, 266Mbp was incorporated into 22 linkage groups in the chromonomed assembly (87% of assembly length) with 550 of the 2104 scaffolds incorporated and five scaffolds having been split. Of those scaffolds not incorporated, the mean length and N50 length were 26Kbp and 4Kbp, respectively, and no markers aligned to over 92% of these 1554 relatively small scaffolds.

### Incorporating conserved gene synteny into the Gulf pipefish integration

**Figure 5:**
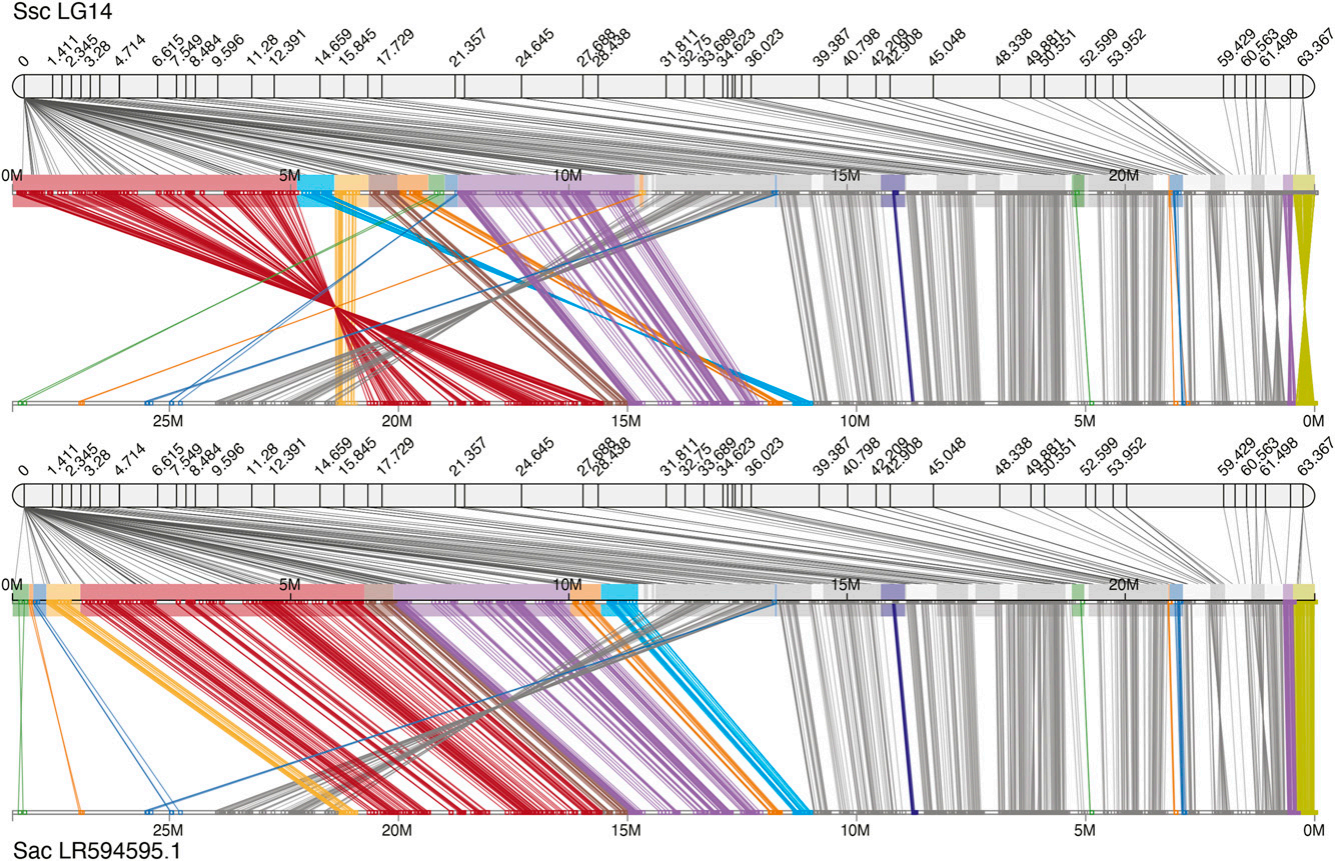
Ortholog-directed scaffold rearrangements in the Gulf pipefish (*Sygnathus scovelli*). Potential improvements in LG14 integrated assembly by incorporation of gene synteny between *S. scovelli* and *S. acus*. Colored scaffolds indicate where synteny was employed, and colors are consistent with Figure 4. In each panel, the *S. scovelli* genetic map is shown above, linking the scaffolds of the physical assembly together. Lines also connect each pair of gene orthologs between *S. scovelli* and *S. acus*.

After the initial incorporation of the genetic map in Gulf pipefish, a number of scaffolds are not entirely resolved, including a large cluster of scaffolds at the top end of linkage group 14 (Figure 4A). Using conserved gene synteny information from a congener, the greater pipefish (*Sygnathus acus)*, Chromonomer was able to order 16 scaffolds and to orient 14 scaffolds (Figure 4B, colored boxes). Figure 5 shows conserved synteny between *S. scovelli* and *S. acus*, before and after Chromonomer employed ortholog-based ordering. Naturally, the process has made *S. scovelli* look more like *S. acus*, which might not always be biologically correct. If the reference organism is sufficiently closely related, however, this method provides a rational hypothesis for a likely order and orientation beyond what was initially arbitrary. This rationale is supported in this case by the fact that many of the reoriented scaffolds display conserved intra-scaffold gene order, and genome-wide there is a strong pattern of conserved synteny between the two pipefish (Fig. S1). Figure 5 also shows that there remain putative true rearrangements between *S. scovelli* and *S. acus*, as demonstrated by Gulf pipefish scaffold 16 in the region at ∼14Mb on LG14 (∼22Mb in *S. acus*). In this case, the scaffold has high support from the genetic map for its position, while the orthologous gene block appears in a different relative location in the *S. acus* genome.

### Rescaffolding an assembly prior to map integration: the platyfish genome

**Figure 6:**
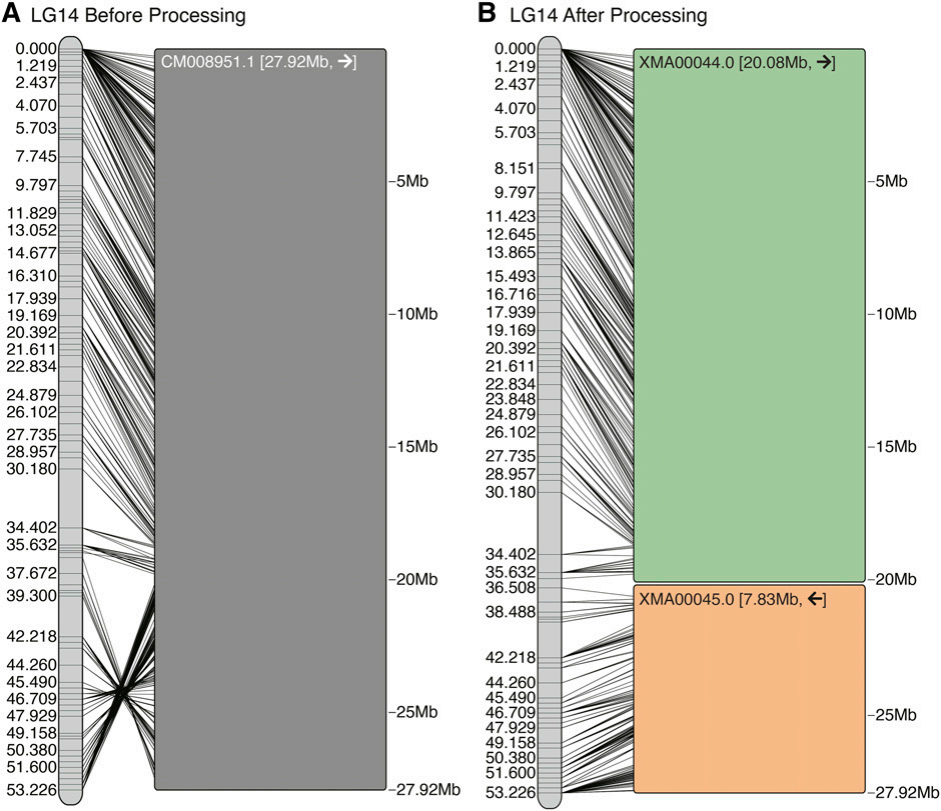
Using virtual gaps and the *rescaffold* algorithm in platyfish (*Xiphophorus maculatus*). (A) The platyfish assembly shows a clear misassembly (an inverted segment between ∼35-53cM) when compared against the genetic map. (B) A consistent order of markers is found on the map, and depth of coverage is employed to split the CM008951.1 contig into 2 components that can then be independently reoriented.

**Figure 7:**
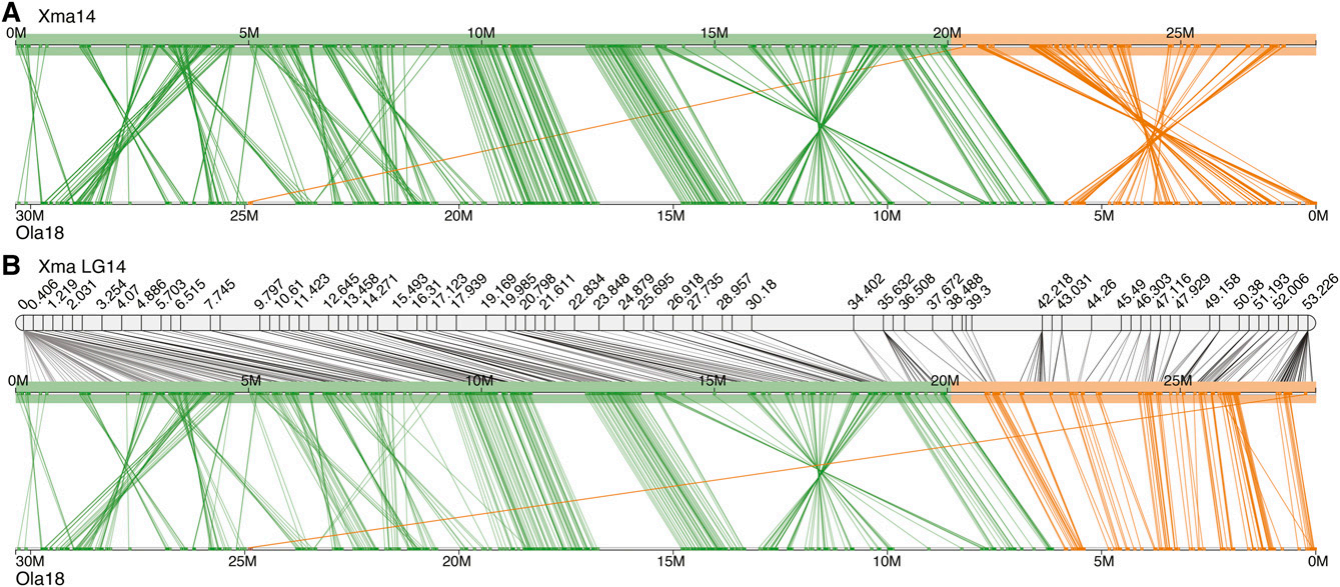
Improvements in the platyfish (*Xiphophorus maculatus*) chromosome-level assembly. Conserved gene synteny between platyfish (Xma) and medaka (*Oryzias latipes*, Ola) illustrates improvements in the LG14 integrated assembly by application of the *rescaffold* algorithm. The top panel (A) shows synteny prior to correction; several inversions are present, including one associated with the platyfish assembly (orange, colored to match the scaffolds in Fig. 6). After correction (B), inversions and ordering are rectified.

As described above, if there is a misassembly that inverts or translocates a component of the contig but does not produce scaffold gaps, the basal Chromonomer algorithm on its own will discard all inconsistent markers but the majority set, leaving the unbroken scaffold at the place in the linkage graph with the largest number of consistent markers. We can see how this would occur in the southern platyfish (*Xiphophorus maculatus*) assembly (Schartl *et al.* 2013). The assembly, prior to the application of Chromonomer, is qualitatively impressive, with 24 chromosome-length contigs, and only an additional 76 scaffolds, with an N50 of 31.5Mbp. The assembly was generated from long-read data, and an optical map was used to further scaffold the assembled contigs (see Table 1 for details). However, when we compare the assembly against a high quality genetic map containing more than 22,000 markers and 267 progeny (a backcross between *X. maculatus* and *X. helleri* (Amores *et al.* 2014)), we find that some putative assembled chromosomes agree strongly with the genetic map (linkage group 1, Fig. S2), but others show potential misassemblies (Figure 6A). A caveat is that, since this genetic map was produced from a hybrid cross, it is possible that some of the conflicts between the assembly and map *could* be due to true differences between the *X. maculatus* and *X. helleri* genomes. The map shows a large inversion relative to the assembly on LG14 between ∼35-53cM, but there exists no clear place for the basal Chromonomer algorithm to break the assembly. Without intervention, Chromonomer would leave the chimeric scaffold in the location with the largest set of correlated markers. We applied Chromonomer’s *rescaffold* option (Figure 2) to prioritize the genetic map order over the contiguous assembly and resolve this situation. Figure 6B shows the result of applying this algorithm to a relatively simple case in the platyfish assembly, where marker order is not cleanly correlated between the genetic map and physical assembly. Comparison of patterns of gene synteny, before and after, relative to the medaka genome (*Oryzias latipes*, Figure 7) illustrates the outcome. The reordered physical assembly is now congruent with gene order in medaka. For an example of more complex corrections employing the rescaffold algorithm in the platyfish assembly, see Figs S3 and S4.

### Algorithmic limits of Chromonomer: the rockcod chromonome

**Figure 8:**
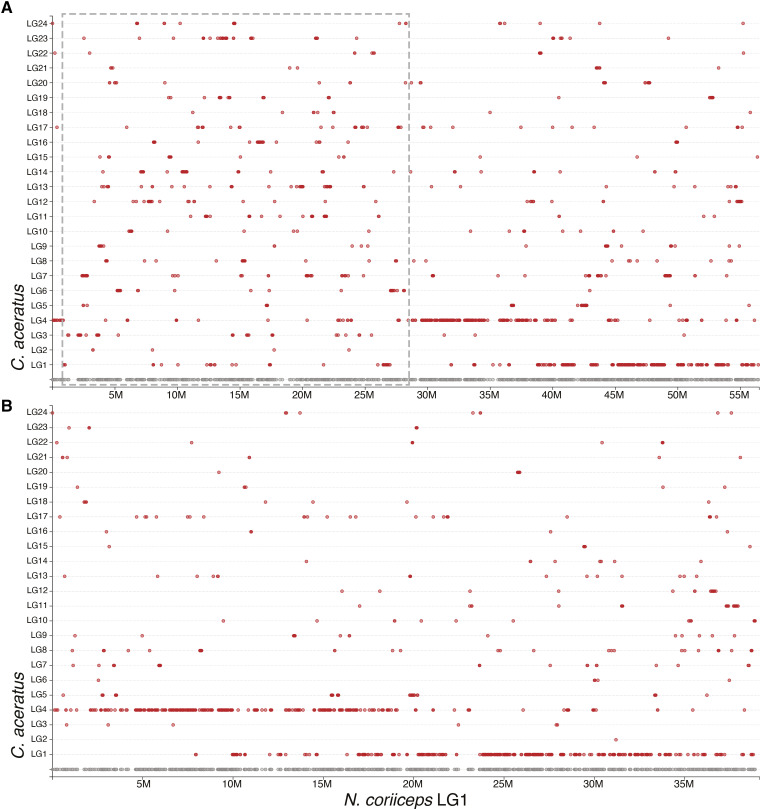
The rockcod (*Notothenia coriiceps*) assembly. All of the large scaffolds in the rockcod assembly appear to be large inter- and intra-chromosomal chimeras. When we examine LG1 in rockcod (A) we can see that orthologous rockcod genes are found scattered across the genome of blackfin icefish, a related taxon. The largest rockcod scaffold, KL668296.1 is highlighted by the dotted line and we can see that it is composed of sizeable pieces from all over the genome. (B) After processing with Chromonomer, the scaffold is broken up and redistributed in the assembly. We can now clearly see the conserved, two-to-one gene synteny between the icefish and rockcod.

**Figure 9:**
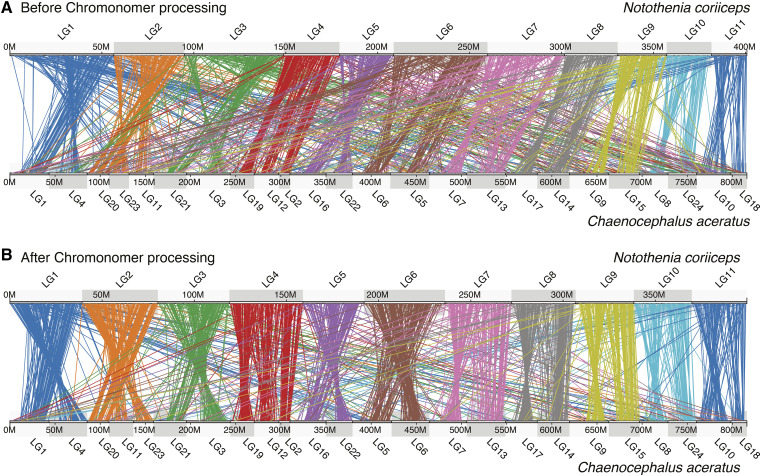
Chromonomer improves the rockcod (*Notothenia coriiceps*) assembly. The rockcod assembly can be chromonomed using the genetic map. (A) shows genome-wide conserved gene synteny prior to integrating the genome. (B) shows marked improvement genome-wide in the assembly after breaking down the largest scaffolds using the genetic map. However, smaller assembly errors remain.

The Antarctic bullhead notothen, or black rockcod (*Notothenia coriiceps*), is an extreme cold-adapted fish with an interesting karyotype. While the ancestral haploid chromosome number in teleost fish is 24 or 25 (Naruse 2004), the black rockcod has just 11 chromosomes (V. P. Prirodina, A. V. Neyelov 1984). Using an outcrossed RADseq-based genetic map constructed from 244 progeny in an F1 pseudo-testcross with 9,138 mappable markers, Amores *et al.* (2017) were able both to confirm this genome evolution occurred by end-to-end fusions and to identify which ancestral chromosomes became fused. The sequenced rockcod genome was also assembled using a hybrid strategy that mixed data from Illumina paired-end libraries, from 454-sequenced mate-pair libraries, and from limited PacBio RS II sequencing (see Table 1 for details). The resulting assembly was composed of 37,605 scaffolds, had a scaffold N50 of 218Kbp, and the largest scaffold was 28.8Mbp in length — a poor result that is not atypical for predominately short-read based assemblies. We used Chromonomer to integrate the physical assembly with the genetic map for the first time. Here we found extreme discordance within the assembled scaffolds. For example, the second largest scaffold, KL668296.1 (27.5Mbp in length), contains 368 markers, but these markers were scattered in the genetic map across every one of the 11 linkage groups (Fig. S5). In fact, the four largest scaffolds map to all 11 linkage groups resulting in a remarkably disordered assembly. The pattern can be seen when gene orthologs are visualized in comparison with a related genome, in Figure 8. The *x*-axis shows genes from rockcod in gray (at bottom) and the corresponding orthologous genes from the blackfin icefish (*Chaenocephalus aceratus*) in red, located on the icefish chromosomes (*y*-axis), but ordered according to the rockcod. Large scaffold KL668296.1 (boxed by a dashed line in Figure 8A) spans nearly half of rockcod linkage group 1, but genes orthologous to those identified on this scaffold are found dispersed all over the icefish genome, a condition unlikely to be biologically true. A multitude of other rockcod scaffolds are also probably chimeric, most likely due to assembly errors that stem from mate-pair libraries, with error amplified by the hybrid assembly and gap closing/scaffold extension algorithms that were optimized for maximal simple statistics (like N50), but not for accuracy. After running the basal algorithm of Chromonomer, scaffold KL668296.1 was broken down into 27 coherent pieces and those were reintegrated into their respective positions according to the genetic map. The resulting increased congruence in conserved gene synteny suggests structural improvement of the assembly (Figure 8B), and importantly, the original signal of chromosome fusion is more cleanly resolved, where a region that is syntenic in rockcod is split between LG1 and LG4 in icefish. If we view the genome-wide conserved gene synteny between rockcod and the blackfin icefish, we see similarly improved synteny, but still a lot of noise. Because of the granular nature of misassembly in this genome, there are not enough markers to fully correct all of the errors, and segments containing single or small numbers of genes remain incorrectly fused to other segments (*e.g.*, Fig. S6); such cases likely account for many of the lines crossing to non-orthologous chromosomes in Figure 9B.

## Discussion

In the decades since the human genome project (International Human Genome Sequencing Consortium 2001) was completed by a large consortium with massive resources (Collins 2003), sequencing technology advances have facilitated order of magnitude improvements to genome assembly though the employment of long-read, high volume sequencers and more advanced scaffolding techniques. However, even with greatly improved N50s, moving a genome from a collection of scaffolds to a chromonome (Braasch *et al.* 2015) with realistic long-range relationships among assembly segments remains a major impediment. Genetic maps are one of the oldest genomic resources, dating back to the beginning of the field of modern genetics (Painter 1933), but by the time of the human genome project they too required significant resources to discover and genotype markers. Short-read, massively parallel sequencing has changed genetics too, as RAD sequencing and software like Stacks made genetic mapping broadly feasible and applicable (Rochette *et al.* 2019). This new generation of genetic mapping provided huge numbers of markers simultaneously with the genotyping itself, and has permitted map building in a single generation. The advantages of marrying these two data streams (genome sequence and genetic map) has been demonstrated in improvement and validation of several recently released reference genomes (International Cassava Genetic Map Consortium (ICGMC) 2015; Kelley *et al.* 2016; Lee *et al.* 2019; Takehana *et al.* 2020; Simakov *et al.* 2020).

There are several pieces of software that aim to integrate genetic maps with genome assemblies. The ALLMAPS software (Tang *et al.* 2015) seeks to optimize the set of markers to maximize concordance between the linkage map positions of markers and their aligned genomic positions. It permutes scaffold positions in the integrated genome to minimize the distance between markers on different scaffolds. ALLMAPS then refines scaffold position and determines orientation using a genetic algorithm. Scaffolds that should be broken are flagged by ALLMAPS but breaking must be done manually. Lep-Anchor (Rastas 2020) also aims to optimize marker order, first employing a Hidden Markov Model to bin markers to different linkage groups and split inter-linkage group scaffolds, and then it uses dynamic programming to calculate the number of markers that support all particular scaffold orders. The Kermit software uses a genetic map to guide a *de novo* assembly (Walve *et al.* 2019). Kermit bins scaffolds given in an initial assembly according to their order in the genetic map, then it places raw, long reads into those bins and creates an overlap graph from that initial order. It completes the assembly based on the sequence in the overlap graph.

In the current work, we have presented Chromonomer, which broadly shares goals with tools like ALLMAPS and Lep-Anchor, however, these software treat integration as an optimization problem; by permuting the set of markers and scaffold positions to generate many different orders, they will discover a best order. This brute-force approach requires that the underlying objects are rational – that is, that they *can* be ordered. Chromonomer is designed around a different philosophy: allow the application of different lines of evidence, and then show where the underlying components fit together and where they do not. Since each assembly and scaffolding strategy brings a particular error model along with it, the key to successfully integrating a physical assembly with a genetic map is the ability to rank the quality of different types of information and to employ the most dependable information in the most rational hierarchy. Given a high-quality assembly, both methods will result in the same, high-quality integrated genome. But given a pathological assembly, the results will be very different. Along these lines, Chromonomer is actually two distinct things: first, a tool to integrate physical and genetic assemblies, and second, a hypothesis generator to be employed during the assembly process itself. In addition, the algorithm Chromonomer applies – modeling each linkage group as a graph – is discrete and does not require optimization, which provides an execution time on the order of minutes (several orders of magnitude faster than optimization-based algorithms, described above (Rastas 2020)).

Integrating the map and the physical assembly can be used powerfully in an iterative process that leads to improvement of the final assembly via improvements in the inputs. Aided by the reporting and visualization tools in Chromonomer, a researcher can improve the genetic map when genotyping errors become obvious in the context of the physical assembly. Similarly, the researcher, when presented with the number and type of scaffold splits conducted by Chromonomer, can choose to re-examine or pare out problematic data types added of the assembly (*e.g.*, a particular sequenced mate-pair library, or the output of software that hybridized different sequenced libraries together) that cause most of the artifacts. In an iterative approach, a researcher can employ knowledge of synteny from multiple species to provide evidence for a particular assembly hypothesis. For example, if a change in gene order coincides with the boundaries of a scaffold, it is likely an assembly error. One of the most innovative applications of Chromonomer is to use it as a tool to compare and contrast different genetic maps, perhaps from members of different populations of the same species, with the same base assembly. Here read depth and synteny information could be combined to explore the nature of structural variants within a species. Treating genomic assembly components (maps, scaffold sets, gene annotations) as independent objects, each providing a different line of evidence, provides a rich informatic framework to explore genome architecture.

Recently, chromosomal conformation capture methods, such as Hi-C, have become very useful in further scaffolding genomes, while versions of optical mapping have become more accessible as well (*e.g.*, BioNano). These methods can be very successful in scaffolding a genome, particularly if combined with long-read-assembled contigs. Do these methods deprecate the use of genetic maps? In our opinion, no. In fact, the approaches complement each other. Chromosomal conformation capture and optical mapping are scaffolding algorithms that can improve assemblies significantly, but they also introduce errors commensurate with the quality of the data. The long-read platyfish assembly shows simple (Figure 6) and complex (Figs. S3) errors resulting from optical map scaffolding. Both scaffolding protocols also rely on high molecular weight DNA and require non-trivial library preparations. On the other hand, genetic maps can provide one of the only independent lines of evidence (a second such line is conserved gene synteny) of the biological correctness of a scaffold, but high-quality maps can be created only in certain organisms and can come with another class of error sources. The highest quality genomes, therefore, will integrate as many of these lines of evidence as possible, including long-molecule methods and genetic maps.

Available assembly and scaffolding software still tend to operate as “black boxes”, with internal algorithmic decisions opaque to the outside user. There are practical reasons for this, including the volume of data involved and difficulty in designating standardized file formats. Genome assembly as a service is also gaining in popularity, which risks further obscuring the underlying nature of a particular genome assembly. Future work in accurate reference genome construction should include software design that exports valuable internal assembly/scaffolding information in common formats, and allows practitioners to use multiple lines of evidence, properly integrated by strong underlying tools, into an evolving assembly hypothesis.
